# Urogenital schistosomiasis infection prevalence targets to determine elimination as a public health problem based on microhematuria prevalence in school-age children

**DOI:** 10.1371/journal.pntd.0009451

**Published:** 2021-06-11

**Authors:** Ryan E. Wiegand, Fiona M. Fleming, Anne Straily, Susan P. Montgomery, Sake J. de Vlas, Jürg Utzinger, Penelope Vounatsou, W. Evan Secor

**Affiliations:** 1 Division of Parasitic Diseases and Malaria, Centers for Disease Control and Prevention, Atlanta, Georgia, United States of America; 2 Swiss Tropical and Public Health Institute, Basel, Switzerland; 3 University of Basel, Basel, Switzerland; 4 SCI Foundation, London, United Kingdom; 5 Department of Public Health, Erasmus MC, University Medical Center Rotterdam, Rotterdam, The Netherlands; Federal University of Ceará, Fortaleza, Brazil, BRAZIL

## Abstract

**Background:**

Recent research suggests that schistosomiasis targets for morbidity control and elimination as a public health problem could benefit from a reanalysis. These analyses would define evidence-based targets that control programs could use to confidently assert that they had controlled or eliminated schistosomiasis as a public health problem. We estimated how low *Schistosoma haematobium* infection levels diagnosed by urine filtration in school-age children should be decreased so that microhematuria prevalence was at, or below, a “background” level of morbidity.

**Methodology:**

Data obtained from school-age children in Burkina Faso, Mali, Niger, Tanzania, and Zambia who participated in schistosomiasis monitoring and evaluation cohorts were reanalyzed before and after initiation of preventive chemotherapy. Bayesian models estimated the infection level prevalence probabilities associated with microhematuria thresholds ≤10%, 13%, or 15%.

**Principal findings:**

An infection prevalence of 5% could be a sensible target for urogenital schistosomiasis morbidity control in children as microhematuria prevalence was highly likely to be below 10% in all surveys. Targets of 8% and 11% infection prevalence were highly likely to result in microhematuria levels less than 13% and 15%, respectively. By contrast, measuring heavy-intensity infections only achieves these thresholds at impractically low prevalence levels.

**Conclusions/significance:**

A target of 5%, 8%, or 11% urogenital schistosomiasis infection prevalence in school-age children could be used to determine whether a geographic area has controlled or eliminated schistosomiasis as a public health problem depending on the local background threshold of microhematuria.

## Author summary

For urogenital schistosomiasis, targets for morbidity control and elimination as a public health problem are based on the percentage of school-age children with a heavy intensity infection in a set of sentinel schools. These targets are not tied to specific morbidity indicators and should be reevaluated due to the recognition that all infections have an impact on people’s health. Multiple studies have shown a strong association between urogenital schistosomiasis infection and microhematuria. In these analyses, data from children aged 6–15 years in monitoring and evaluation cohorts from five African countries were used to determine whether infection and heavy intensity infection targets could be developed, based on a prevalence of microhematuria in a school without schistosomiasis infections before and after initiation of a deworming program. Results indicate that targets of 5%, 8%, or 11% urogenital schistosomiasis infection prevalence in school-age children can be used to reliably conclude that a school is below a microhematuria prevalence of 10%, 13%, or 15%, respectively. These targets could be used by control program managers as guide to determine whether morbidity has been controlled or eliminated as a public health problem.

## Introduction

The primary goal of the World Health Organization (WHO) schistosomiasis control program guidelines since the early 2000s has been to reduce morbidity [1]. For most of sub-Saharan Africa, the primary control intervention currently employed is the distribution of praziquantel as preventive chemotherapy [2]. Due to limited praziquantel availability when the guidelines were developed, WHO focused on reducing the most severe morbidities caused by schistosomiasis. Based on limited data showing associations between severe morbidities and heavy intensity infections (≥50 *Schistosoma haematobium* eggs per 10 ml of urine) [3], control of morbidity was defined as a <5% prevalence of heavy intensity infections (PHI) and elimination of schistosomiasis as a public health problem was defined as <1% PHI [4]. Recent analyses suggest the prevalence of morbidity associated with these PHI targets for morbidity control and elimination as a public health problem may not be distinguishable from each other [5], drawing into question their programmatic usefulness. Furthermore, WHO guidance for frequency of preventive chemotherapy is based on infection prevalence [1]. This results in most country programs only measuring prevalence and creates a disconnect between the stated program goals and implementation of interventions to achieve them.

Praziquantel donations from Merck KGaA have increased the number of people treated globally nearly 10-fold from 2006 to 2017 [6]. Concurrently, there has been an increased appreciation of more subtle manifestations of schistosomiasis morbidity [7–9]. Thus, there is interest in refocusing schistosomiasis guidelines on morbidity control [2] with calls to create more detailed and specific targets [10]. A robust, evidence-based target that identifies the point at which schistosomiasis is eliminated as a public health problem will be crucial to meet the goal to approximately double the number of countries validated as having eliminated schistosomiasis as a public health problem by 2023 [11].

Microhematuria is strongly associated with *S*. *haematobium* infection and has been used in place of egg detection to estimate community-level prevalence [12]. A meta-analysis of the use of dipstick-detected microhematuria for the diagnosis of *S*. *haematobium* infection found 81% (95% confidence interval (CI) 79–83%) sensitivity and 89% (95% CI 87–92%) specificity across different locations and subgroups [13]. Analyses of data from multiple, national schistosomiasis control programs found microhematuria prevalence correlated well with PHI [5] and WHO’s infection intensity categories [14]. Therefore microhematuria would appear to be a strong candidate as a morbidity indicator that can be reduced to a threshold level that is locally acceptable [11].

Because identifying an indicator for eliminating schistosomiasis as a public health problem is critical to the success of control programs for the next 10 years [11], we investigated *S*. *haematobium* infection prevalence levels and their likelihood to be the same as or lower than “background” microhematuria levels, i.e., the prevalence of microhematuria in an area with no cases of schistosomiasis. We utilized literature on the expected background prevalence of microhematuria [12,15] to determine potential threshold levels. Evaluations were performed using control program data before and after one and two years of annual preventative chemotherapy with praziquantel from five African countries supported through the Schistosomiasis Control Initiative (SCI) [16].

## Methods

### Ethics statement

The Imperial College Research Ethics Committee (ICREC_8_2_2, EC No. 03.36, R&D No. 03/SB/003E) and each country’s ethical review boards located in the Ministries of Health approved the use of these data. The US Centers for Disease Control and Prevention (CDC) was determined to be a non-engaged research partner. Meetings were held with teachers and parents to inform them about participation in these programs. Verbal consent from community leaders in Mali was given since this was the standard consent at the time. For the other countries, head teachers provided written informed consent. Parents or guardians provided consent and children provided assent. Patients and the public were not involved in the design, conduct, reporting, or dissemination plans of this research.

### Study design and data collection

Data for these analyses come from national control programs for schistosomiasis and soil-transmitted helminthiasis in Burkina Faso [17,18], Mali [18], Niger [18], Tanzania [19], and Zambia [19]. Briefly, countries began a national program scale-up including distribution of praziquantel (for schistosomiasis) and albendazole (for soil-transmitted helminthiasis) to treat endemic populations based on WHO guidance [20]. Two monitoring and evaluation cohorts were used in these analyses. First, a longitudinal cohort of children aged 6–12 years in primary schools that were randomly selected from various endemic settings. Schools were treated every year and followed for two more years with the following exceptions: Burkina Faso did not treat schools between baseline and follow-up 1; some schools in Mali were treated twice between baseline and follow-up 1 and some schools were treated annually but no data were collected at follow-up 1; Tanzanian schools were not treated between follow-up 1 and follow-up 2; and Zambia did not collect data after follow-up 1. When a school failed to be treated once annually, that school was removed from subsequent surveys. We also included any children aged 6–15 years from a second community cohort and pooled with the children from the longitudinal cohort for school-level estimates. The second cohort mostly contained adults, but had a range of persons aged 4–88 years.

Both cohorts were sampled for the same number of years at approximately the same time. These surveys were performed approximately one year apart and are referred to as baseline, follow-up 1, and follow-up 2. Participants were required to be above 94 cm in height and not currently ill. Praziquantel and albendazole were distributed to consenting participants each year. The baseline survey occurred pre-treatment; the two follow-up surveys each occurred immediately before the annual praziquantel dose.

### Infection and microhematuria data

The presence of *S*. *haematobium* was determined by urine filtration. School-age children from Burkina Faso, Tanzania, and Zambia were evaluated with a single urine sample and single filtration; Nigerien school-age children were evaluated by two filtrations from a single urine sample. In Mali, school-age children were evaluated with single filtrations of two urine samples collected on consecutive days. Approximately 10 ml of urine were passed through a filter, stained, and microscopically examined for *S*. *haematobium* eggs. If any eggs were detected in a urine sample, that participant was deemed positive. School-age children with 50 or more *S*. *haematobium* eggs per 10 ml of urine were deemed to have a heavy intensity infection. Microhematuria was assessed with Hemastix (Bayer Diagnostics; Basingstoke, United Kingdom) dipsticks according to manufacturer instructions. School-age children with a positive or trace-positive (hemolyzed or non-hemolyzed) reading were considered positive.

### Data analysis methods

R version 4.0.3 was used for analyses. A binomial, Bayesian, errors in variables [21,22] model was used to assess the relationship between infection and microhematuria prevalence. This approach accounts for uncertainty in the prevalence of microhematuria and prevalence of infection by assuming both are binomially distributed. For each microhematuria prevalence threshold, infection measurement (all infection prevalence or PHI), and survey, four models were fit. The first three models used linear, quadratic, and cubic functions of infection prevalence, respectively, to predict morbidity prevalence. The fourth used a function utilized by van der Werf and de Vlas [23] for associating community infection prevalence and morbidity indicator prevalence. Models were fit via Markov chain Monte Carlo (MCMC) methods using the rjags package [24]. Full details on the statistical and MCMC methods are included in the S1 Text along with results from all models. Best fitting models were determined by the smallest deviance information criterion (DIC) [25] (Table A in S1 Text).

We explored three microhematuria prevalence thresholds. Thresholds of 13% and 15% were based on the results of Krauth and colleagues [12] who found the prevalence of microhematuria unrelated to *S*. *haematobium* infection to be approximately 13% or 15–20% after incorporating data from a systematic review [15]. They estimated about 3.4% of those infections to be missed infections post-treatment, suggesting 10% could be a more appropriate estimate.

Results are presented as the percent chance that a school’s *S*. *haematobium* infection prevalence had a microhematuria prevalence less than the threshold of 10%, 13%, or 15%. A depiction of the relationship between infection prevalence, microhematuria prevalence, and the percent chance is included in Figure A in S1 Text. From these curves, one chooses an appropriate percent chance to determine the infection prevalence. The highest infection prevalence that is predicted to result in a microhematuria prevalence below the morbidity threshold is then referred to as the target prevalence. The same approach was used for modeling PHI, where the results are reported as the percent chance that a school with a given PHI target is below the threshold.

## Results

A total of 91 schools were analyzed at baseline, 41 at follow-up 1, and 22 at follow-up 2. The median (and range) for *S*. *haematobium* infection prevalence was 44.4% (0.0–99.0%) at baseline, 11.3% (0.0–83.5%) at follow-up 1, and 31.2% (2.8–89.2%) at follow-up 2. For microhematuria prevalence, the median (and range) was 38.5% (0.8–94.7%) at baseline, 11.3% (0.0–60.4%) at follow-up 1, and 14.8% (4.0–71.6%) at follow-up 2.

**Fig 1:**
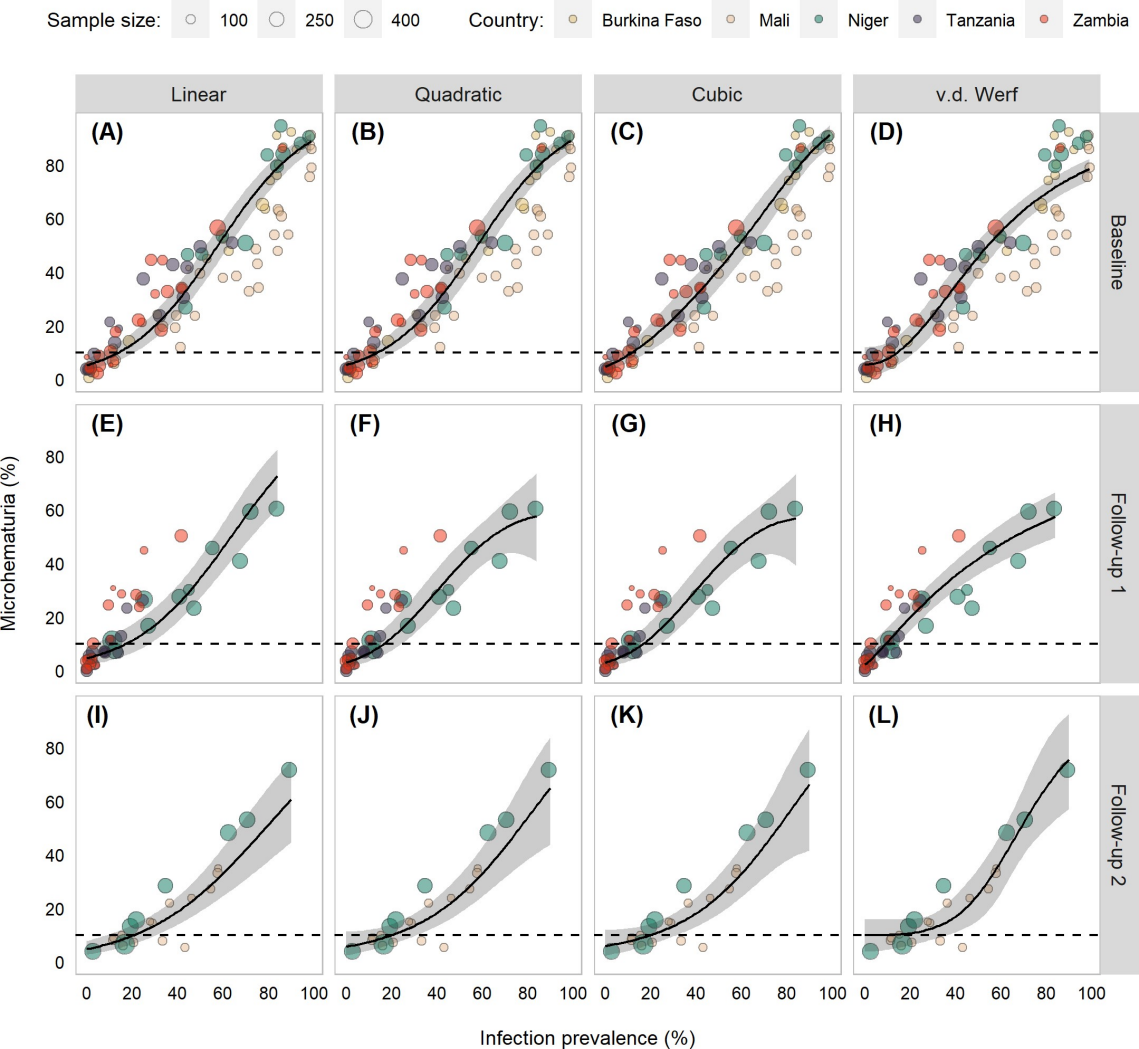
Scatter plots of *Schistosoma haematobium* infection prevalence and microhematuria prevalence of children aged 6–15 years from Burkina Faso, Mali, Niger, Tanzania, and Zambia participating in schistosomiasis control program activities between 2003 and 2008. Points are scaled based on the number of school-age children with *S*. *haematobium* infection data. Black line indicates model fits and gray shading indicates 95% Bayesian credible intervals (BCIs) from errors in variables model. The dashed line denotes 10% microhematuria prevalence.

A grid of scatter plots with each model fit shows only slight differences in the models (Fig 1), which was also true when considering the fit statistics (Table A in S1 Text) and the model predictions in the range most relevant to these analyses (Fig B in S1 Text).

**Fig 2:**
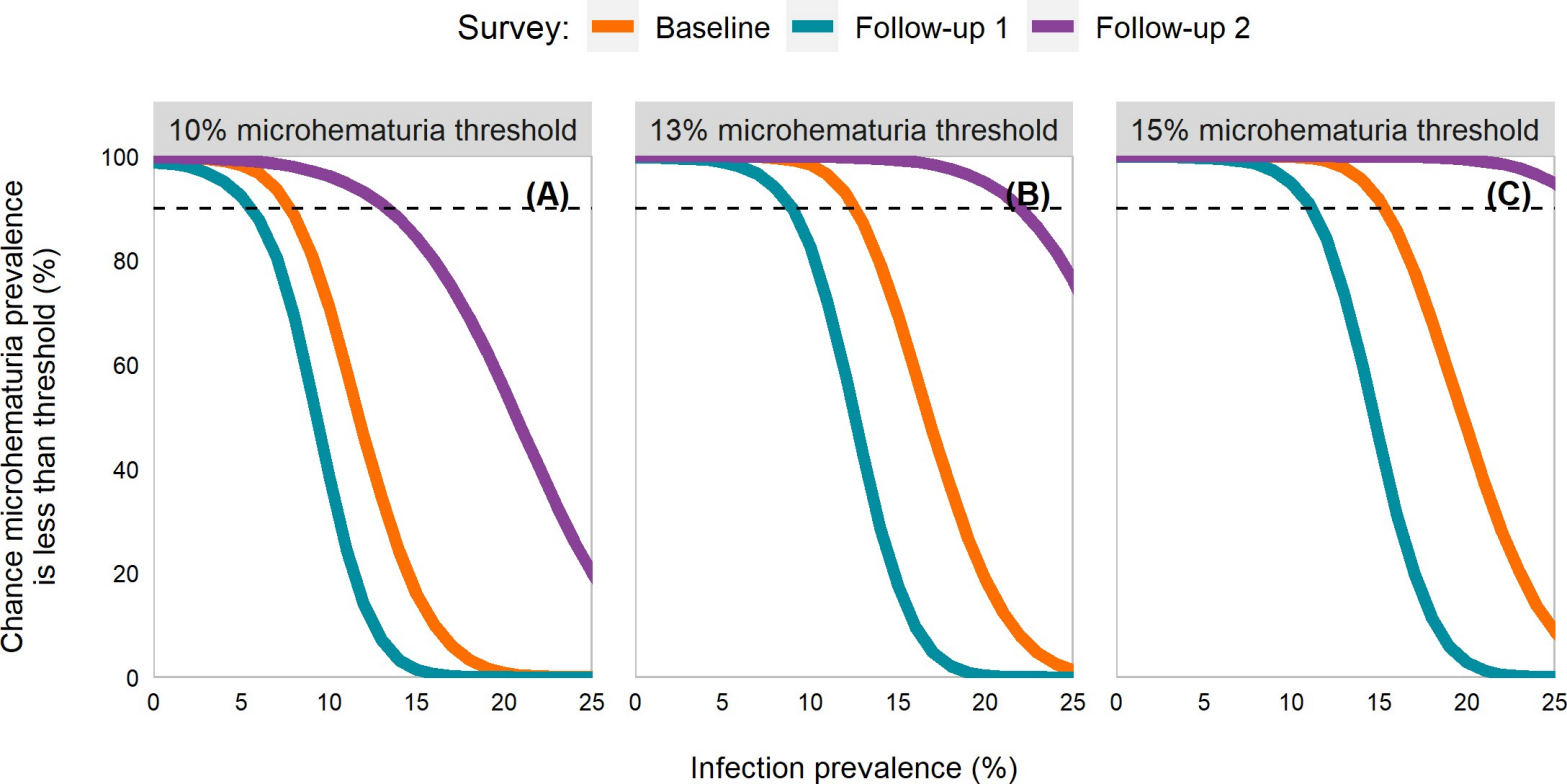
Line plots by survey of the percent chance a school with a given *Schistosoma haematobium* infection prevalence is below the microhematuria threshold. Panel A is for a 10% microhematuria threshold; panel B for 13%; and panel C for 15%. Estimates utilized children aged 6–15 years from Burkina Faso, Mali, Niger, Tanzania, and Zambia participating in schistosomiasis control program activities between 2003 and2008. Full description of the methods is in S1 Text. The dashed line indicates a 90% chance of being below the threshold.

Best fitting curves of infection prevalence and the percent chance of a school falling below the three microhematuria thresholds were largely similar across surveys, with the baseline curve falling between the two follow-up curves (Fig 2). Curves from all models were largely consistent, though at follow-up 2 the function utilized by van der Werf and de Vlas [23] failed to reach a 90% chance of achieving the microhematuria threshold (Fig C in S1 Text). In best fitting models, infection prevalence targets ≤5%, 8%, and 11% provided a 90% chance that the microhematuria thresholds of 10%, 13%, and 15%, respectively, would be met. The 8% and 11% were very close to having a 95% chance of reaching the 10% and 15% thresholds, respectively, but the percent chance estimate at follow-up 1 was slightly less than 95% (94.0% for the 8% target and 94.8% for the 11% target in Table B in S1 Text).

**Fig 3:**
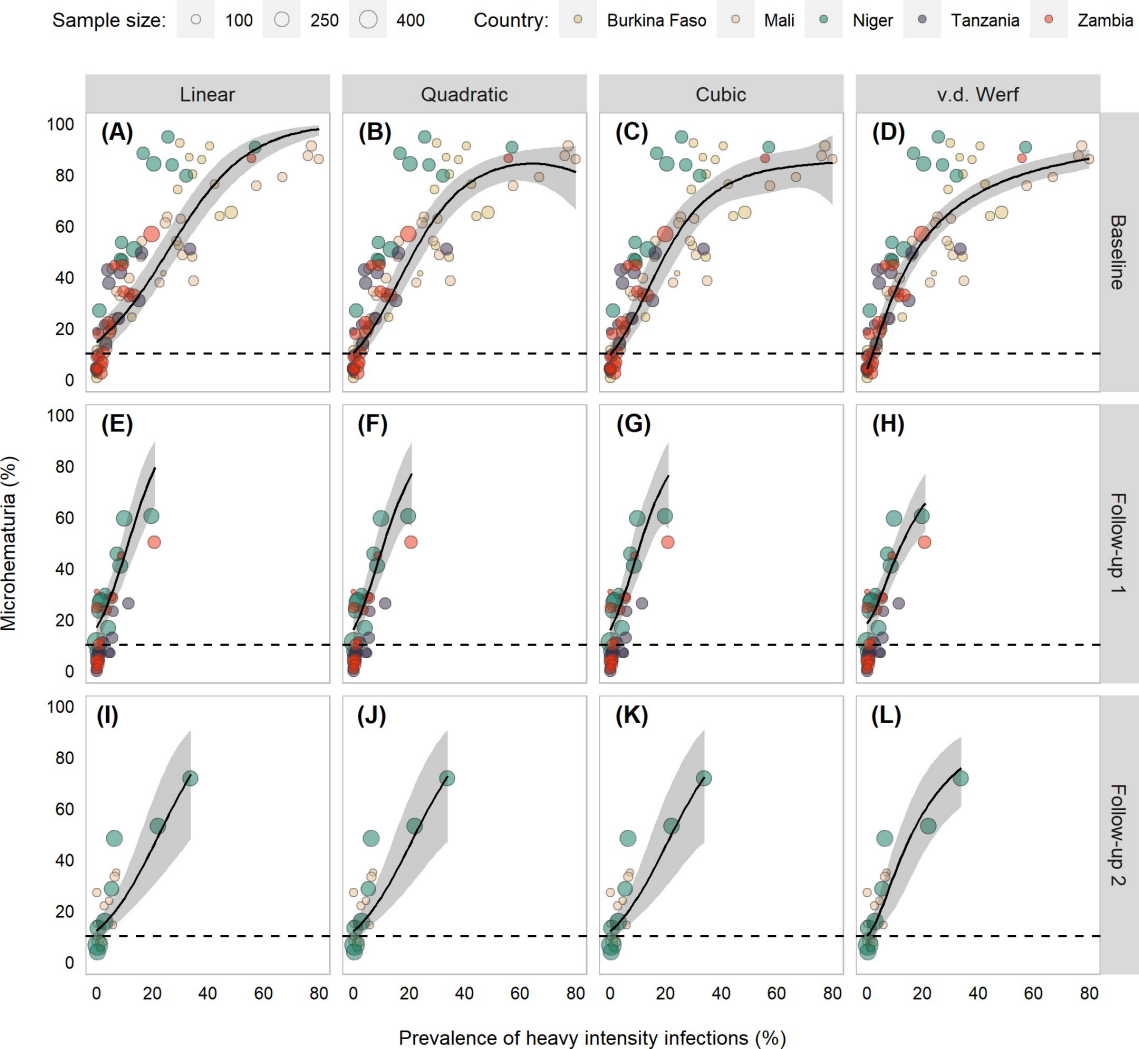
Scatter plots of *Schistosoma haematobium* prevalence of heavy intensity infections (PHI) and microhematuria prevalence of children aged 6–15 years from Burkina Faso, Mali, Niger, Tanzania, and Zambia participating in schistosomiasis control program activities between 2003 and 2008. Points are scaled based on the number of school-age children with *S*. *haematobium* infection data. Black line indicates model fits and gray shading indicates 95% Bayesian credible intervals (BCIs) from errors in variables model. The dashed line denotes 10% microhematuria prevalence.

**Fig 4:**
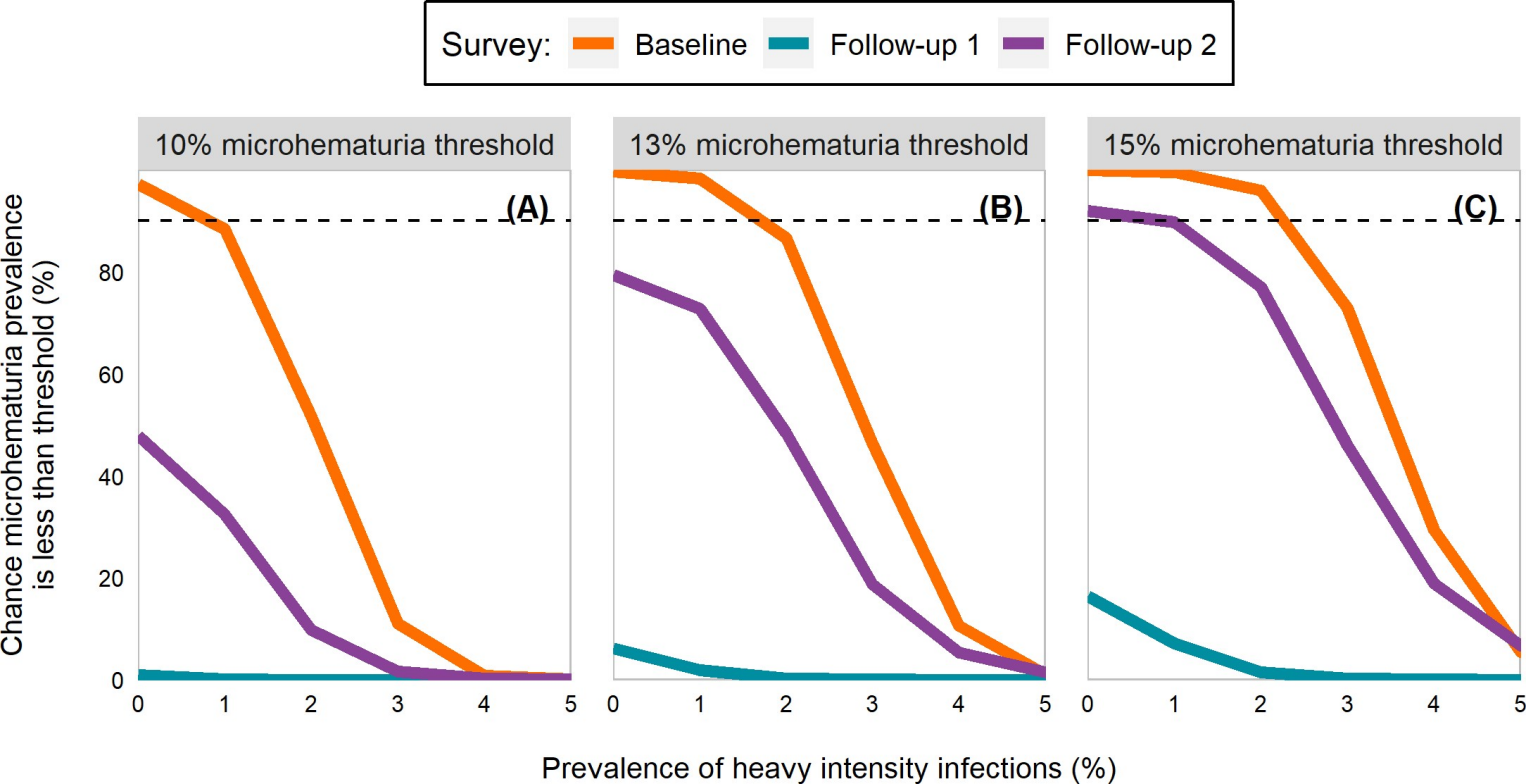
Line plots by survey of the percent chance a school with a given *Schistosoma haematobium* prevalence of heavy intensity infections is below the microhematuria threshold. Panel A is for a 10% microhematuria threshold, panel B for 13%, and panel C for 15%. Estimates utilized children aged 6–15 years from Burkina Faso, Mali, Niger, Tanzania, and Zambia participating in schistosomiasis control program activities between 2003 and 2008. Full description of the methods is in S1 Text. The dashed line indicates a 90% chance of being below the threshold.

The median and range of PHI in schools studied was 9.1% (0.0–79.8%) at baseline, 1.5% (0.0–20.7%) at follow-up 1, and 1.8% (0.0–33.8%) at follow-up 2. Model fits differed some at the low end (Fig D in S1 Text) and high end (Fig 3) at baseline but were largely similar for the follow-up surveys. At baseline, PHI targets of 0%, 1%, and 2% provided a 97.2%, 98.3%, and 96.0% chance, respectively, that the microhematuria thresholds of 10%, 13%, and 15% would be met (Fig 4; Table C in S1 Text). At follow-up 1, no PHI-based target was able to achieve above a 16.4% chance of reaching any of the thresholds. At follow-up 2, a PHI target of 0% was estimated to have a 92.0% chance of reaching the 15% threshold.

## Discussion

Our study examined the relationship between microhematuria and *S*. *haematobium* infection prevalence. The results suggest different infection prevalence targets that are realistic and measurable for evaluating whether microhematuria has reached, or passed below, a background level threshold and therefore schistosomiasis is eliminated as a public health problem. Based on these results, national control programs could choose one of the three microhematuria thresholds (10%, 13%, or 15%) and a percent chance of achieving it, to determine an infection prevalence target based on the follow-up survey results. We presented targets of 5%, 8%, and 11% infection prevalence since these had a high (90%) chance that they would result in a school possessing a microhematuria prevalence below the thresholds of 10%, 13%, and 15%, respectively. Though, programs could choose a different certainty. For instance, a 15% threshold with an 80% chance of achieving the threshold suggests an infection prevalence target of ≤ 12%.

The results were largely consistent across the three surveys, which gave us confidence in the robustness of these target values. The consistency was especially true at the lower prevalence targets when certainty was high. This could be due to the inclusion of schools from five countries with different ecologic archetypes, thus, giving us a broad representation of different endemic areas. There were some differences between the baseline and follow-up surveys. For instance, as the infection prevalence target increased at follow-up 1, the percent chance tended to decrease much quicker. At a 9% infection prevalence target, the percent chance of falling below the 10% microhematuria threshold was 81% at baseline and 97.2% at follow-up 2 but only 54.5% at follow-up 1. Typically, the follow-up 2 survey consistently had higher certainty. Some of this difference could be attributed to changes in the distributions of infection and microhematuria prevalence post-preventive chemotherapy administration, though bias may have been introduced since many schools were lost to follow-up or excluded due to not being treated once in the preceding year. The heterogeneity between schools was much larger at baseline since far more schools had higher infection prevalence and microhematuria percentages (Fig 1).

By contrast, PHI-derived targets provided minimal adaptability and could be prone to misclassification. For 10% microhematuria, there was at least a 90% chance that a school was below the morbidity threshold only at baseline, when PHI was 0%. No prevalence could achieve a 99% chance of meeting the 10% threshold. This includes the current WHO target of 1% PHI, which was unable to reach a 90% chance of meeting the 10% threshold in any survey. For baseline and follow-up 2, there was a steep drop in the percent chance of meeting the threshold usually after 2%, suggesting a small number of missed heavy-intensity infections could easily cause a misclassification.

The overarching goal of this study was to discover readily measurable and well-defined program targets for urogenital schistosomiasis in school-age children. An evidence-based target is crucial for schistosomiasis programs to achieve elimination as a public health problem, which is the stated goal of the WHO 2021–2030 neglected tropical disease (NTD) Roadmap [11]. Other NTDs have based their validation process around countries falling below defined prevalence targets [26,27]. Defined targets are necessary for program managers as they allow them to determine the efficacy of control efforts, identify when problematic issues such as poor coverage or infection "hotspots" occur, or when to consider switching goals from elimination as a public health problem to interruption of transmission. Current PHI-based targets are unable to ensure that microhematuria is reduced to a background level and are highly sensitive to small changes, whereas the targets proposed here appear robust and can be adapted to different situations.

Our results are specific to *S*. *haematobium* infections in school-age children and may not be appropriate for older age groups. However, because the prevalence and intensity of schistosome infections are highest in this population and most national programs are based on preventive chemotherapy of school-age children, we believe these targets provide great value for schistosomiasis morbidity control. Furthermore, because the more severe manifestations of schistosomiasis morbidity result from multiple years of infection, effective programs for this school-age group will eventually reduce *S*. *haematobium* infection-associated morbidity in older individuals as well. For example, Kenyan adults who had received preventive chemotherapy as children demonstrated an 11-fold reduction of bladder wall pathologies compared to previously untreated individuals despite having similar infection levels as adults [28].

Our results are limited by a few factors. There are individual-level factors which influence the background level of microhematuria, such as the age of first menses in girls. Our assumption is that evaluations of morbidity control and elimination as a public health problem are done at an aggregate level and that a random sample, i.e., one that draws children across sexes and ages, is ascertained. The effect of deviations from a random sample should be studied so the effect on the evaluation is known. The level of certainty of our results may vary by location due to the wide variability of microhematuria prevalence previously noted [12]. This could be due to measurement error or variations in the presence of other concomitant phenomena such as menses or concurrent conditions such as urinary tract infections [13,23]. An evaluation’s sample size and design could also play a role in an appropriate target. The target may also depend on the dipstick manufacturer utilized. Data in these analyses used Hemastix dipsticks, the same that were used in the study by Krauth and colleagues [12]. These results are also not immediately applicable to programs focused on control of *S*. *mansoni* and new research is still needed to identify a robust morbidity indicator for intestinal schistosomiasis species [5,14]. In addition, further work will be necessary to identify target infection levels in other age groups as morbidity can manifest differently in older individuals with more chronic infections. Efforts to identify species-specific morbidity markers are underway [29].

We believe our modeling approach provides a useful framework for defining morbidity-related targets. This modeling approach can be utilized for other diseases which need to set a target based on a morbidity marker. For schistosomiasis, this method can be used again when a suitable intestinal schistosomiasis marker is identified or when improved diagnostics become available and targets need to be recalibrated. Since different thresholds can be evaluated simultaneously and the Bayesian methodology allows for the computation of probability-based measures, this allows for targets to be computed for locations based on program needs.

## Conclusion

These analyses present empirically-based infection targets for urogenital schistosomiasis that utilize the association between infection prevalence and microhematuria prevalence. Our findings can be used by schistosomiasis control programs as targets to reduce microhematuria prevalence below an established background level, and could be adopted to meet the goals in the current road map [11]. Current targets based on PHI are not set low enough for control of schistosomiasis-related microhematuria and cannot be reliably used by control programs for this purpose.

## Supporting information

S1 Text Supporting information including all additional tables and figures. (DOCX)
