# Supplementary material for: Urogenital schistosomiasis infection prevalence targets to determine elimination as a public health problem based on microhematuria prevalence in school-age children
Source: PLoS Negl Trop Dis. 2021 Jun 11;15(6):e0009451. doi: 10.1371/journal.pntd.0009451 (PMC8221785; doi:10.1371/journal.pntd.0009451)
Supplement: S1 Text — (DOCX) [file pntd.0009451.s001.docx]

Supplementary materials for: “Urogenital schistosomiasis infection prevalence targets to determine elimination as a public health problem based on microhematuria prevalence in school-age children”

Ryan E. Wiegand^1,2,3*^, Fiona M. Fleming^4^, Anne Straily^1^, Susan P. Montgomery^1^, Sake J. de Vlas^5^, Jürg Utzinger^2,3^, Penelope Vounatsou^2,3^, W. Evan Secor^1^

1. Division of Parasitic Diseases and Malaria, Centers for Disease Control and Prevention, Atlanta, Georgia, United States of America
2. Swiss Tropical and Public Health Institute, Basel, Switzerland
3. University of Basel, Basel, Switzerland
4. SCI Foundation, London, United Kingdom
5. Department of Public Health, Erasmus MC, University Medical Center Rotterdam, Rotterdam, The Netherlands

Contents

[Statistical Methods 3](#_Toc72169344)

[Overview 3](#_Toc72169345)

[Model Details 3](#_Toc72169346)

[Model Implementation 5](#_Toc72169347)

[Overview 5](#_Toc72169348)

[JAGS model code for cubic function 6](#_Toc72169349)

[JAGS model code for van der Werf and colleagues’ function 7](#_Toc72169350)

[Supplemental Tables 8](#_Toc72169351)

[Table A. Deviance information criterion (DIC) values for all models fit. Bold font indicates lowest DIC value for a dataset and infection prevalence measure (any infection or heavy-intensity infection). 8](#_Toc72169352)

[Table B. Estimated percent chance of falling below 10%, 13% and 15% microhematuria thresholds for infection prevalence targets between 0 and 25% percent. Values in this table are plotted in Figure 2. 9](#_Toc72169353)

[Table C. Estimated percent chance of falling below 10%, 13% and 15% microhematuria thresholds for heavy intensity infection prevalence (PHI) targets between 0 and 5% percent. Values in this table are plotted in Figure 4. 10](#_Toc72169354)

[Supplemental Figures 11](#_Toc72169355)

[Figure A. Posterior distributions of each infection prevalence at the baseline survey, as determined by a cubic model, with shading for the proportion of the distribution which falls above and below the microhematuria threshold of 10%. Blue indicates the proportion below the threshold of 10% and red indicates the proportion that is above the threshold. The medians correspond to the curve in panel C of Figure B. 11](#_Toc72169356)

[Figure B. Focused version of Figure 2 with only model fits confined to the range 0-20% infection intensity prevalence. Best fitting models (see Table A for DIC values which were used to determine best fitting) are represented by thicker line and shaded bands representing 95% credible intervals. 12](#_Toc72169357)

[Figure C. Line plots of the percentage chance a school, with a given *Schistosoma haematobium* infection prevalence, will fall below a microhematuria threshold for all models considered. Thresholds of 10%, 13%, and 15% were considered. Estimates utilized children age 6-15 years from Burkina Faso, Mali, Niger, Tanzania, and Zambia participating in schistosomiasis control program activities between 2003-2008. Predictions were based on errors in variable Bayesian models. Models were fit separately for each survey. 13](#_Toc72169358)

[Figure D. Focused version of Figure 4 with only model fits confined to the range 0-5% prevalence of heavy intensity infections. Best fitting models are represented by thicker line and shaded bands representing 95% credible intervals. 14](#_Toc72169359)

[Figure E. Line plots of the percentage chance a school, with a given *Schistosoma haematobium* prevalence of heavy intensity infection, will fall below a microhematuria threshold for all models considered. Thresholds of 10%, 13%, and 15% were considered. Estimates utilized children age 6-15 years from Burkina Faso, Mali, Niger, Tanzania, and Zambia participating in schistosomiasis control program activities between 2003-2008. Predictions were based on errors in variable Bayesian models. Models were fit separately for each survey. 15](#_Toc72169360)

[Bibliography 16](#_Toc72169361)

# Statistical Methods

## Overview

The goal of this modeling is to identify a *Schistosoma haematobium* infection target or heavy-intensity infection prevalence target where microhematuria prevalence is kept below a level that is assumed to represent when schistosomiasis is eliminated as a public health problem.

In these analyses, the term “threshold” refers to the microhematuria prevalence which is assumed to represent when schistosomiasis is eliminated as a public health problem. From previous research [1, 2], 10%, 13%, or 15% where chosen as potential targets. The term “target” defines the *S. haematobium* infection prevalence or prevalence of heavy intensity infection (PHI), where the percent chance of reaching the target is above an accepted percent change (or probability). In these analyses, all integer infection prevalence values from 0% to 100% were considered.

Since each school or community will have different numbers of people ascertained, a binomial distribution was a natural choice for describing the variation in microhematuria prevalence. Since the prevalence of infection or PHI will also follow a binomial distribution, an errors in variables model [3, 4] was used. Bayesian methods were chosen, since direct estimates of the percent chance that microhematuria prevalence falls below the target is desired.

## Model Details

We aggregated all individual data to the school or community level. This largely concurs with programmatic schistosomiasis evaluations since usually no individual level covariates are included in analyses. If the inclusion of individual level covariates is desired, then a different modeling approach is needed. Nevertheless, with data aggregated to the school or community level, we assume the frequency of the morbidity indicator, *y*, is binomially distributed in the following way

$$y_{i}\sim Bn(n_{i},p_{i})$$

where *i* denotes a school or community, $n_{i}$ is the number of participants evaluated for the morbidity, and $p_{i}$ is the probability a participant has microhematuria. The probability of microhematuria is assumed to be Beta distributed where

$$p_{i}\sim\text{Beta}\left( \phi\mu_{i},\phi\left[ 1-\mu_{i} \right] \right).$$

The parameter $\phi$ is used to control the spread of the Beta distribution. Based on testing with the microhematuria data, $\phi$ was given a $\text{Gamma(}\text{20,1)}$ prior for all analyses. Finally, $\mu_{i}$is the estimate from the standard logit transform. For the cubic model,

$$\mu_{i}\text{=}\frac{e^{\beta_{o}+\beta_{1}r_{i}+\beta_{2}{r_{i}}^{2}+\beta_{3}{r_{i}}^{3}+\beta_{4_{1}}\text{country}_{1}+\cdots+\beta_{4_{k}}\text{country}_{k}}}{{1+e}^{\beta_{o}+\beta_{1}r_{i}+\beta_{2}{r_{i}}^{2}+\beta_{3}{r_{i}}^{3}+\beta_{4_{1}}\text{country}_{1}+\cdots+\beta_{4_{k}}\text{country}_{k}}}$$

where the $\beta$’s are estimated by the model and all have Cauchy prior distributions with a center of zero and a scale of 2.5.[5] $\beta_{0}$ is the intercept and the others are the coefficients for each power of the predictor variable, $r_{i}$. $r_{i}$is the true infection risk of participants with a *S. haematobium* infection (or when heavy-intensity infections are used, the proportion of participants with a heavy intensity infection). The quadratic and linear functions utilize the same approach but remove the cubic (for quadratic) and cubic and quadratic (for linear) terms to estimate $\mu_{i}$. Indicator variables are included for each of the *k* countries included in each analysis. The observed frequency $x_{i}$ is the number of participants with an *S. haematobium* infection in school or community *i* where a total of $m_{i}$ participants were evaluated for *S. haematobium*. Thus,

$$x_{i}\sim B(m_{i},r_{i})$$

and $r_{i}$ has $\mathcal{U}\text{(0,1)}$ distribution. The inclusion of $r_{i}$ instead of using $\frac{x_{i}}{m_{i}}$ allows the proportion to vary in the Markov Chain Monte Carlo (MCMC) simulations according to the size of $m_{i}$.

We also used a function utilized by van der Werf and colleagues[6-8] to associate community prevalence of *S. haematobium* with the prevalence of morbidity indicators, specifically

$$\mu_{i}=\frac{a+b*{r_{i}}^{c}+\beta_{1}\text{country}_{1}+\cdots+\beta_{k}\text{country}_{k}}{1+b{*r_{i}}^{c}},$$

where $0\leq a\leq1$,$b>0$ and $c>1$. In this function, $a$ is the morbidity prevalence due to other diseases, and $b$ and $c$ describe the association between $r_{i}$ and $\mu_{i}$. Prior distributions for these parameters followed these bounds where $a\sim\mathcal{U}\text{(0}\text{.}\text{001,1)}$, $b\sim\mathcal{U}\text{(0}\text{.}\text{01,100)}$, $c\sim\mathcal{U}\text{(1}\text{.}\text{01,100)}$, and $\beta_{j}\sim Cauchy(0, 2.5)$. As before, $\mu_{i}$ is used to estimate the proportion of participants with the morbidity in question through the Beta distribution.

# Model Implementation

## Overview

Models were fit via MCMC using JAGS [9] and CODA [10] in R via the rjags package.[11] Three chains were fit with an adaptive phase of 20,000 iterations per chain. The iterations from the adaptive phase were discarded and each chain was run for another 100,000 iterations.

After completing the 100,000 iterations, graphical displays of the trace and densities functions were used to determine if any chains or a subset of iterations should be discarded. The deviance information criterion (DIC), as defined by Plummer[12], was used to evaluate model fit and the model with the smallest value was chosen as the final model (Table A). Predicted values are calculated as the median of the posterior distribution and 95% credible intervals are the 2.5% and 97.5% percentiles of the posterior distribution. Finally, we calculated the percentage of empirically sampled values below the microhematuria threshold at each chosen infection prevalence target. A graphical example of this calculation is included in the supplementary figures for infection prevalence thresholds with a threshold of 10% (Fig A). These are the posterior distributions from a cubic model of infection prevalence from the baseline survey. Each infection prevalence has its own posterior distribution and the shading indicates the percentage of the distribution that is below the threshold of 10% (blue) and the proportion that is above the threshold of 10% (red). The more the distribution is colored blue, the greater the likelihood that a school at that infection prevalence will fall below the threshold. The solid black lines at the middle of the distribution indicate the median. If the points at the bottom of the median line were connected, this is the curve that appears in panel C of Fig C and shows the connection between the plots in Fig B and C to Fig 1.

## JAGS model code for cubic function

model{

for (i in 1:N){

# Likelihood

x[i] ~ dbinom(r[i], m[i])

r[i] ~ dunif(0, 1)

y[i] ~ dbinom(p[i], n[i])

p[i] ~ dbeta(alpha[i], beta[i]) T(0.001,0.999)

alpha[i] <- phi * mu[i]

beta[i] <- phi * (1 - mu[i])

logit(mu[i]) <- b[1] + b[2] * r[i] + b[3] * r[i]^2 + b[4] * r[i]^3 + inprod(cty[1:Q], X[i,1:Q])

resid[i] <- y[i]/n[i] - p[i]

residp[i] <- step(resid[i])

}

# priors

for (f in 1:P) {

b[f] ~ dt(0, pow(2.5,-2), 1)

}

for (g in 1:Q) {

cty[g] ~ dt(0, pow(2.5,-2), 1)

}

phi ~ dgamma(20, 1)

# predicted prevalence and exceedance prob

for (j in 1:n.pred.x) {

pred[j] <- ilogit(b[1] + b[2] * pred.x1[j] + b[3] * pred.x2[j] + b[4] * pred.x3[j])

exprob10[j] <- step(.1-pred[j])

}

}

## JAGS model code for van der Werf and colleagues’ function

model{

for (i in 1:N){

# Likelihood

x[i] ~ dbinom(r[i], m[i])

r[i] ~ dunif(0, 1)

y[i] ~ dbinom(p[i], n[i])

p[i] ~ dbeta(alpha[i], beta[i]) T(0.001,0.999)

alpha[i] <- phi * mu[i]

beta[i] <- phi * (1 - mu[i])

mu[i] <- (a + b * (r[i]^c) + inprod(cty[1:Q], X[i,1:Q])) / (1 + b * (r[i]^c))

resid[i] <- y[i]/n[i] - p[i]

residp[i] <- step(resid[i])

}

# priors

a ~ dunif(0.001, 1)

b ~ dunif(0.01, 1)

c ~ dunif(1.01, 10)

for (g in 1:Q) {

cty[g] ~ dt(0, pow(2.5,-2), 1)

}

phi ~ dgamma(20, 1)

# predicted prevalence and exceedance prob

for (j in 1:n.pred.x) {

pred[j] <- (a + b * (((j-1)/100)^c)) / (1 + b * (((j-1)/100)^c))

exprob10[j] <- step(.1-pred[j])

}

}

# Supplemental Tables

## Table A. Deviance information criterion (DIC) values for all models fit. Bold font indicates lowest DIC value for a dataset and infection prevalence measure (any infection or heavy-intensity infection).

| Model | Linear | Quadratic | Cubic | v.d. Werf |
| --- | --- | --- | --- | --- |
| Microhematuria and any infection | | | | |
| Baseline | 1146.871 | 1147.571 | **1143.903** | 1164.832 |
| Follow up 1 | 502.5347 | 498.2412 | 497.9989 | **497.8581** |
| Follow up 2 | **287.6194** | 287.669 | 287.883 | 288.2922 |
| Microhematuria and heavy-intensity infection | | | | |
| Baseline | 1115.552 | 1108.235 | 1106.894 | **1098.904** |
| Follow up 1 | 447.5615 | 446.4221 | 446.3236 | **441.1899** |
| Follow up 2 | 245.8518 | 245.6101 | 245.6344 | **242.9806** |

## Table B. Estimated percent chance of falling below 10%, 13% and 15% microhematuria thresholds for infection prevalence targets between 0 and 25% percent. Values in this table are plotted in Figure 2.

|  | 10% microhematuria threshold | | | 13% microhematuria threshold | | | 15% microhematuria threshold | | |
| --- | --- | --- | --- | --- | --- | --- | --- | --- | --- |
| Infection Prevalence (%) | Baseline | Follow up 1 | Follow up 2 | Baseline | Follow up 1 | Follow up 2 | Baseline | Follow up 1 | Follow up 2 |
| 0 | 99.92 | 98.81 | 99.83 | 100 | 99.84 | 100 | 100 | 99.97 | 100 |
| 1 | 99.86 | 98.51 | 99.77 | 100 | 99.79 | 100 | 100 | 99.95 | 100 |
| 2 | 99.75 | 97.91 | 99.69 | 100 | 99.69 | 100 | 100 | 99.93 | 100 |
| 3 | 99.54 | 96.88 | 99.58 | 100 | 99.54 | 99.99 | 100 | 99.89 | 100 |
| 4 | 99.12 | 95.14 | 99.41 | 99.99 | 99.30 | 99.99 | 100 | 99.83 | 100 |
| 5 | 98.31 | 92.35 | 99.21 | 99.98 | 98.86 | 99.99 | 100 | 99.70 | 100 |
| 6 | 96.69 | 87.84 | 98.90 | 99.95 | 98.02 | 99.98 | 100 | 99.51 | 100 |
| 7 | 93.71 | 80.51 | 98.50 | 99.88 | 96.56 | 99.97 | 100 | 99.15 | 100 |
| 8 | 88.77 | 69.30 | 97.95 | 99.69 | 94.03 | 99.95 | 99.99 | 98.46 | 100 |
| 9 | 81.28 | 54.50 | 97.19 | 99.26 | 89.77 | 99.93 | 99.96 | 97.17 | 100 |
| 10 | 71.02 | 38.50 | 96.19 | 98.35 | 82.67 | 99.89 | 99.85 | 94.81 | 100 |
| 11 | 58.91 | 24.45 | 94.83 | 96.39 | 71.90 | 99.85 | 99.61 | 90.73 | 99.99 |
| 12 | 46.16 | 13.97 | 93.10 | 92.85 | 57.97 | 99.77 | 99.07 | 83.97 | 99.98 |
| 13 | 34.35 | 7.14 | 90.82 | 87.20 | 42.75 | 99.67 | 97.89 | 73.66 | 99.97 |
| 14 | 24.19 | 3.31 | 87.98 | 79.27 | 28.61 | 99.49 | 95.54 | 60.36 | 99.96 |
| 15 | 16.03 | 1.41 | 84.38 | 69.31 | 17.46 | 99.24 | 91.60 | 45.58 | 99.93 |
| 16 | 10.07 | 0.57 | 80.05 | 58.32 | 9.60 | 98.88 | 85.74 | 31.37 | 99.90 |
| 17 | 5.97 | 0.20 | 74.92 | 46.97 | 4.80 | 98.35 | 77.81 | 19.81 | 99.85 |
| 18 | 3.34 | 0.06 | 69.01 | 36.25 | 2.20 | 97.58 | 68.40 | 11.36 | 99.75 |
| 19 | 1.74 | 0.02 | 62.41 | 26.68 | 0.95 | 96.48 | 58.21 | 5.91 | 99.61 |
| 20 | 0.84 | 0.01 | 55.23 | 18.70 | 0.38 | 94.93 | 47.62 | 2.84 | 99.37 |
| 21 | 0.38 | 0 | 47.70 | 12.42 | 0.14 | 92.80 | 37.33 | 1.27 | 99.01 |
| 22 | 0.17 | 0 | 40.20 | 7.85 | 0.05 | 89.96 | 28.04 | 0.53 | 98.49 |
| 23 | 0.08 | 0 | 32.86 | 4.65 | 0.01 | 86.29 | 20.09 | 0.21 | 97.67 |
| 24 | 0.03 | 0 | 26.10 | 2.57 | 0 | 81.67 | 13.65 | 0.07 | 96.49 |
| 25 | 0.01 | 0 | 20.05 | 1.31 | 0 | 76.08 | 8.81 | 0.02 | 94.77 |
|  |  |  |  |  |  |  |  |  |  |

## Table C. Estimated percent chance of falling below 10%, 13% and 15% microhematuria thresholds for heavy intensity infection prevalence (PHI) targets between 0 and 5% percent. Values in this table are plotted in Figure 4.

|  | 10% microhematuria threshold | | | 13% microhematuria threshold | | | 15% microhematuria threshold | | |
| --- | --- | --- | --- | --- | --- | --- | --- | --- | --- |
| PHI (%) | Baseline | Follow up 1 | Follow up 2 | Baseline | Follow up 1 | Follow up 2 | Baseline | Follow up 1 | Follow up 2 |
| 0 | 97.24 | 0.86 | 47.82 | 99.67 | 6.20 | 79.42 | 99.94 | 16.44 | 91.97 |
| 1 | 88.34 | 0.11 | 32.41 | 98.27 | 1.82 | 72.74 | 99.63 | 7.13 | 89.71 |
| 2 | 51.58 | 0 | 9.69 | 86.58 | 0.23 | 48.37 | 95.96 | 1.49 | 77.07 |
| 3 | 10.94 | 0 | 1.57 | 46.35 | 0.01 | 18.80 | 72.88 | 0.17 | 45.98 |
| 4 | 0.80 | 0 | 0.24 | 10.52 | 0 | 5.37 | 29.32 | 0.01 | 18.89 |
| 5 | 0.02 | 0 | 0.05 | 1.03 | 0 | 1.52 | 5.42 | 0 | 6.74 |

# Supplemental Figures

## Figure A. Posterior distributions of each infection prevalence at the baseline survey, as determined by a cubic model, with shading for the proportion of the distribution which falls above and below the microhematuria threshold of 10%. Blue indicates the proportion below the threshold of 10% and red indicates the proportion that is above the threshold. The medians correspond to the curve in panel C of Figure B.


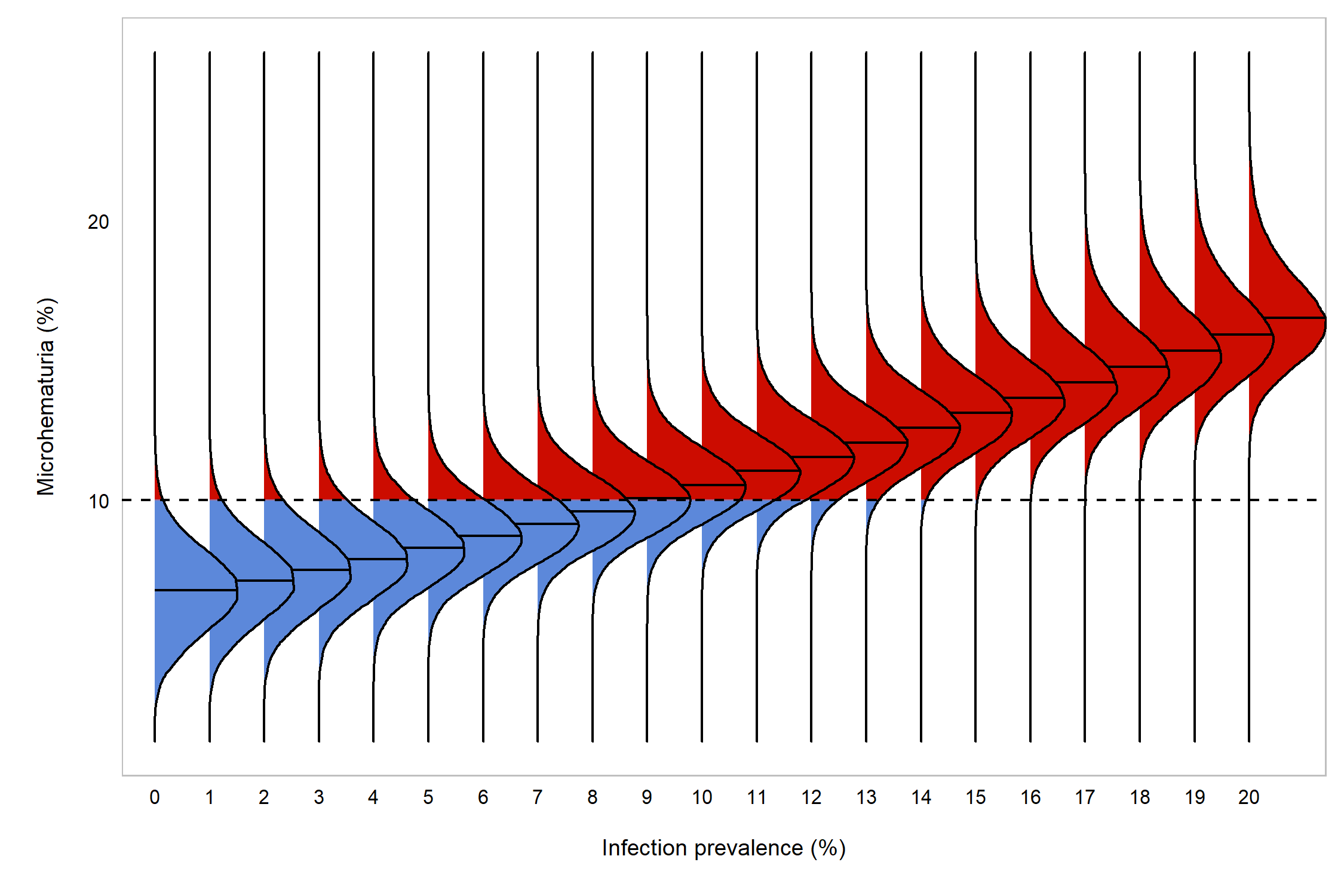


## Figure B. Focused version of Figure 2 with only model fits confined to the range 0-20% infection intensity prevalence. Best fitting models (see Table A for DIC values which were used to determine best fitting) are represented by thicker line and shaded bands representing 95% credible intervals.


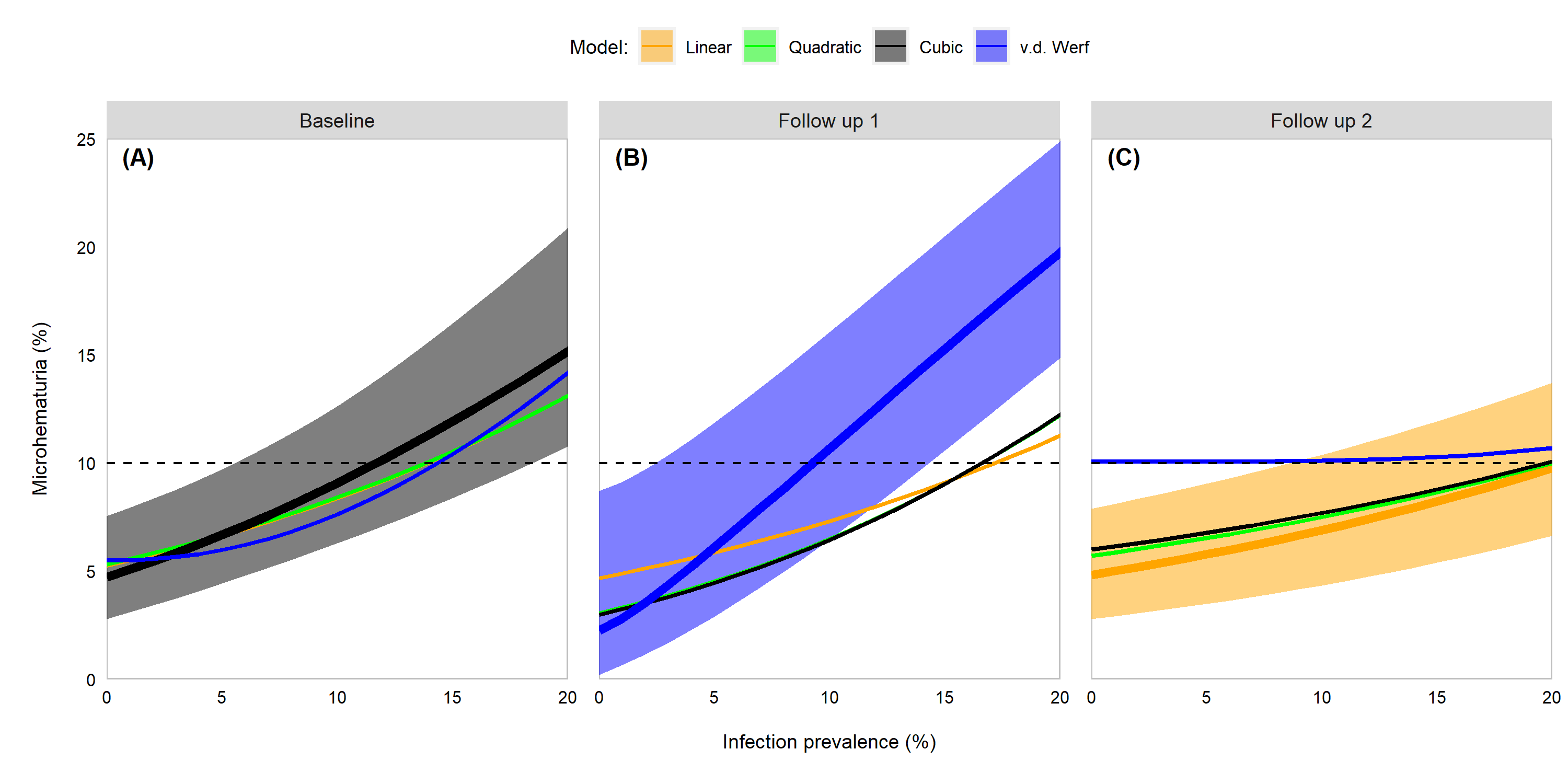


## Figure C. Line plots of the percentage chance a school, with a given *Schistosoma haematobium* infection prevalence, will fall below a microhematuria threshold for all models considered. Thresholds of 10%, 13%, and 15% were considered. Estimates utilized children age 6-15 years from Burkina Faso, Mali, Niger, Tanzania, and Zambia participating in schistosomiasis control program activities between 2003-2008. Predictions were based on errors in variable Bayesian models. Models were fit separately for each survey.


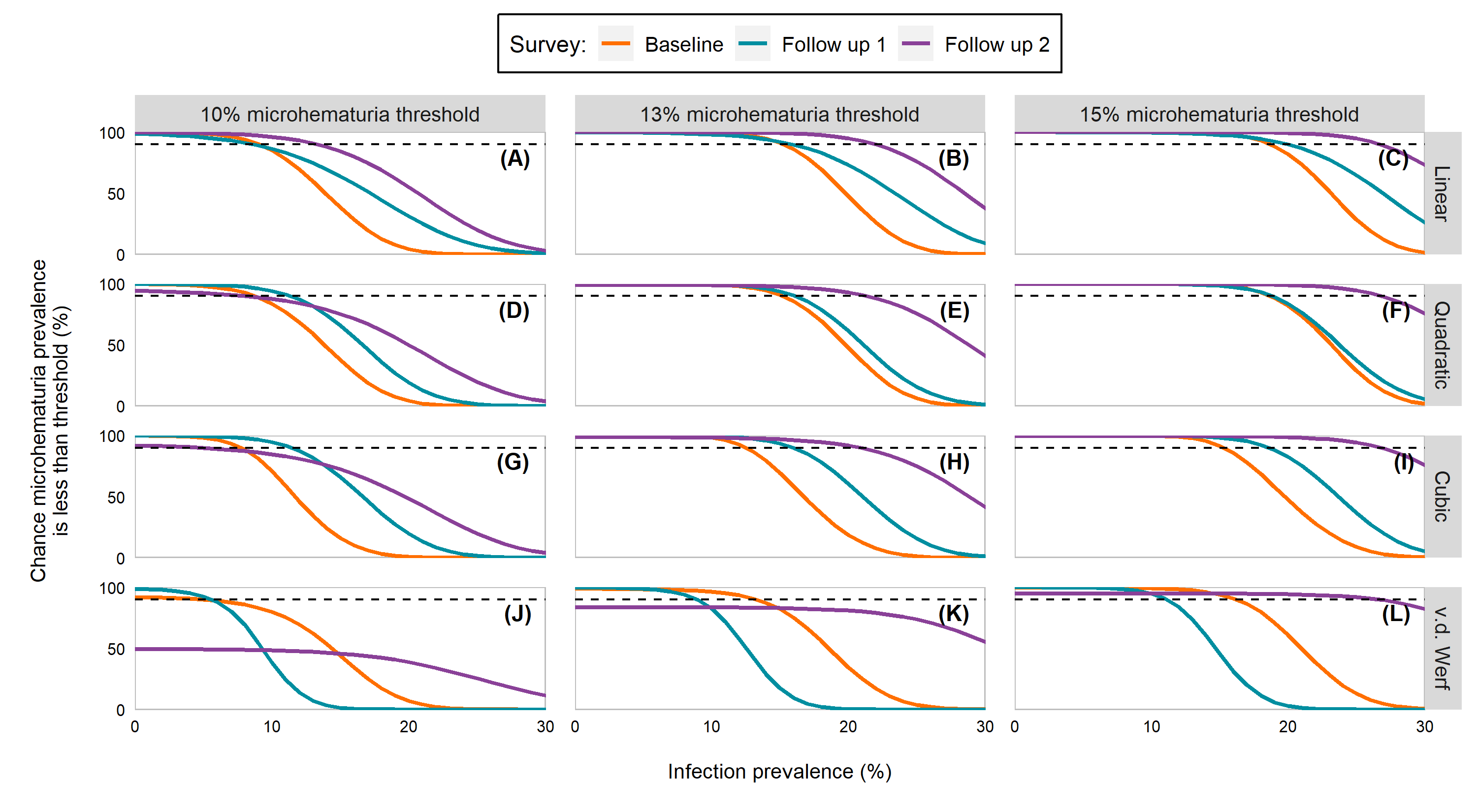


## Figure D. Focused version of Figure 4 with only model fits confined to the range 0-5% prevalence of heavy intensity infections. Best fitting models are represented by thicker line and shaded bands representing 95% credible intervals.


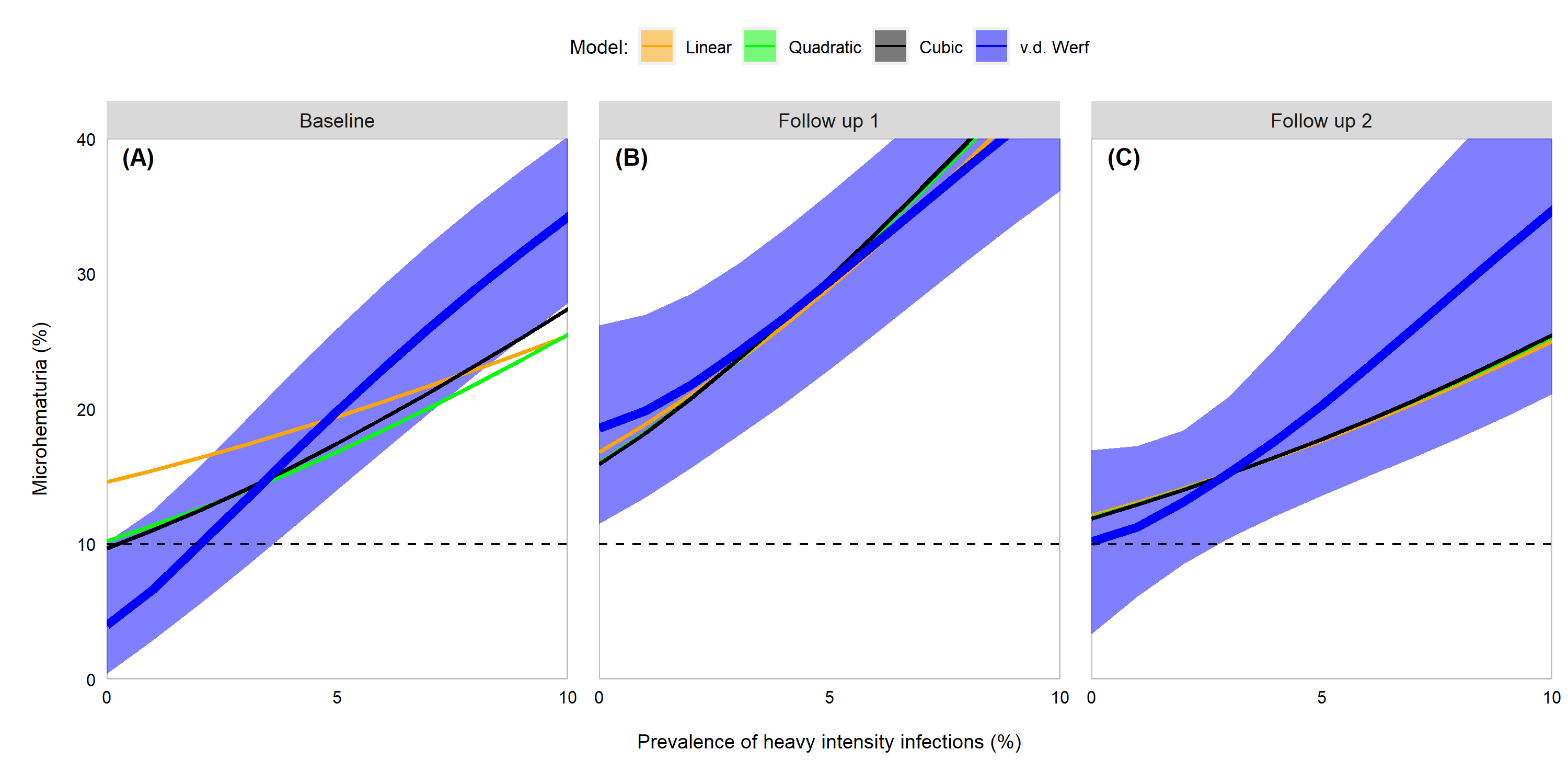


## Figure E. Line plots of the percentage chance a school, with a given *Schistosoma haematobium* prevalence of heavy intensity infection, will fall below a microhematuria threshold for all models considered. Thresholds of 10%, 13%, and 15% were considered. Estimates utilized children age 6-15 years from Burkina Faso, Mali, Niger, Tanzania, and Zambia participating in schistosomiasis control program activities between 2003-2008. Predictions were based on errors in variable Bayesian models. Models were fit separately for each survey.


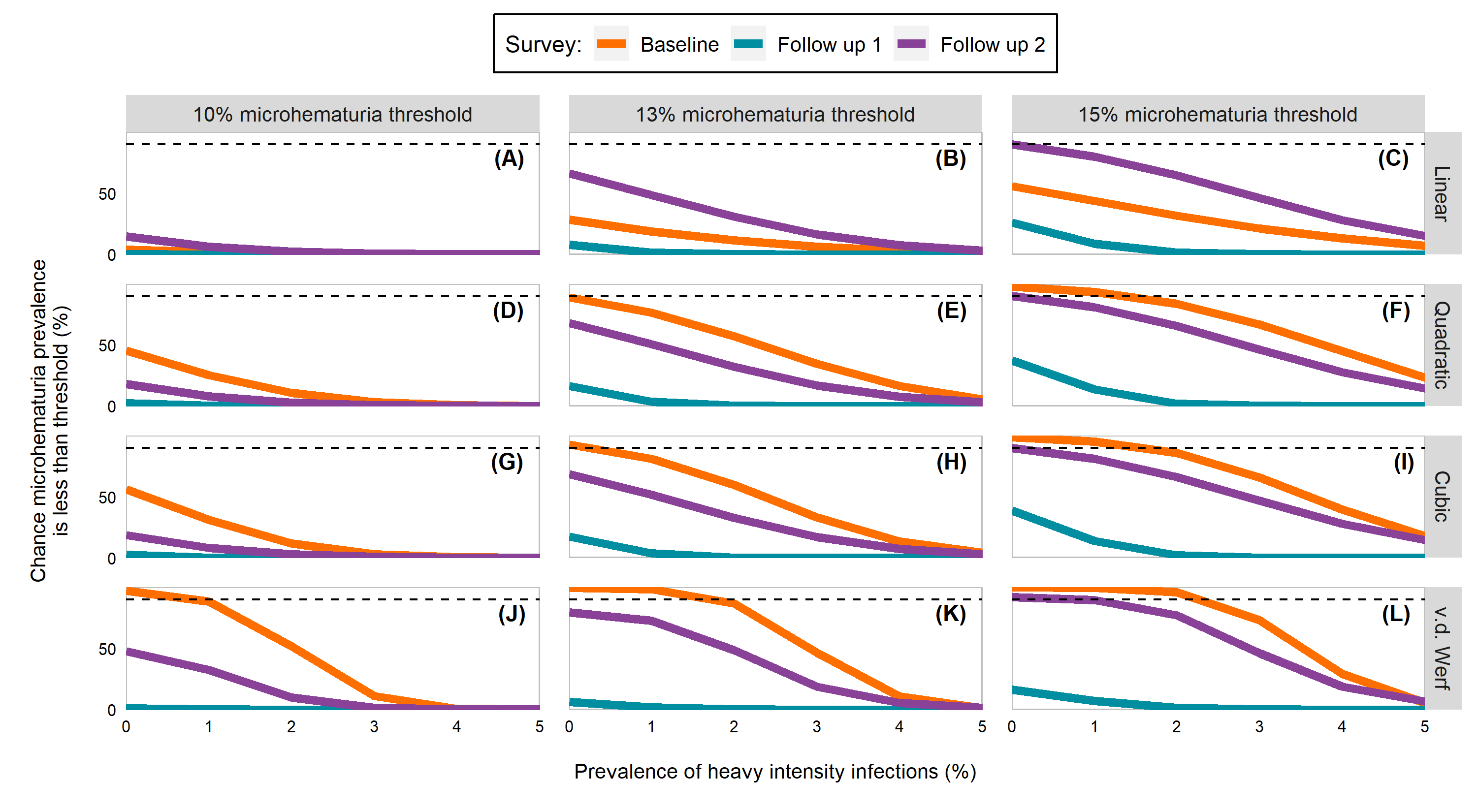


# Bibliography

1. Krauth SJ, Greter H, Stete K, Coulibaly JT, Traoré SI, Ngandolo BNR, et al. All that is blood is not schistosomiasis: experiences with reagent strip testing for urogenital schistosomiasis with special consideration to very-low prevalence settings. Parasite Vector. 2015;8(1):584. doi: 10.1186/s13071-015-1165-y.

2. Ochodo EA, Gopalakrishna G, Spek B, Reitsma JB, van Lieshout L, Polman K, et al. Circulating antigen tests and urine reagent strips for diagnosis of active schistosomiasis in endemic areas. Cochrane Db Syst Rev. 2015;(3):CD009579. doi: 10.1002/14651858.CD009579.pub2. PubMed PMID: CD009579.

3. Durbin J. Errors in variables. Rev Inst Int Stat. 1954;22(1/3):23-32. doi: 10.2307/1401917.

4. Carroll RJ, Spiegelman CH, Lan KKG, Bailey KT, Abbott RD. On errors-in-variables for binary regression models. Biometrika. 1984;71(1):19-25. doi: 10.1093/biomet/71.1.19.

5. Gelman A, Jakulin A, Pittau MG, Su Y-S. A weakly informative default prior distribution for logistic and other regression models. Ann Appl Stat. 2008;2(4):1360-83. doi: 10.1214/08-AOAS191.

6. van der Werf MJ, de Vlas SJ, Looman CW, Nagelkerke NJ, Habbema JD, Engels D. Associating community prevalence of *Schistosoma mansoni* infection with prevalence of signs and symptoms. Acta Trop. 2002;82(2):127-37. Epub 2002/05/22. PubMed PMID: 12020885.

7. van der Werf MJ, de Vlas SJ, Brooker S, Looman CW, Nagelkerke NJ, Habbema JD, et al. Quantification of clinical morbidity associated with schistosome infection in sub-Saharan Africa. Acta Trop. 2003;86(2-3):125-39. Epub 2003/05/15. PubMed PMID: 12745133.

8. van der Werf MJ, de Vlas SJ. Diagnosis of urinary schistosomiasis: a novel approach to compare bladder pathology measured by ultrasound and three methods for hematuria detection. Am J Trop Med Hyg. 2004;71(1):98-106. doi: <https://doi.org/10.4269/ajtmh.2004.71.98>.

9. Plummer M, editor. JAGS: A program for analysis of Bayesian graphical models using Gibbs sampling. Vienna, Austria: Austrian Science Foundation; 2003.

10. Plummer M, Best N, Cowles K, Vines K. CODA: convergence diagnosis and output analysis for MCMC. R news. 2006;6(1):7-11.

11. Plummer M. rjags: Bayesian Graphical Models using MCMC. R package version 4-10 ed2019.

12. Plummer M. Discussion of the paper by Spiegelhalter et al. J Roy Stat Soc B Met. 2002;64(4):620-1. doi: 10.1111/1467-9868.00353.
